# Send nudis: An assessment of nudibranch diversity in Sodwana Bay, South Africa

**DOI:** 10.1002/ece3.10676

**Published:** 2023-11-02

**Authors:** L. Garner, C. J. Oosthuizen

**Affiliations:** ^1^ Department of Zoology and Entomology University of Pretoria Pretoria South Africa

**Keywords:** bioindicators, climate change, diversity, nudibranch, reef health, Sodwana Bay

## Abstract

Climate change is posing unprecedented pressure onto marine ecosystems worldwide. This makes it imperative to monitor the effects that are being experienced in these environments. Nudibranchs are benthic marine organisms that possess characteristics that have the potential to act as indicators of change within ecosystems such as coral reefs. Therefore, these species have the ability to provide valuable information on fine‐scale changes in environmental conditions. It is thus essential for studies, such as this, to establish baseline analyses from which changes within nudibranch populations can be examined in order to investigate their ability to act as bioindicators. Recommendations can also be made for future sampling procedures through investigating environmental and experimental parameters that influence nudibranch communities. Nudibranch populations were sampled on Two‐Mile Reef in Sodwana Bay, South Africa, through SCUBA where individuals were photographed and later identified. Data were collected within a sample‐based dataset, as well as by citizen scientists within an incidence‐based dataset. Across both datasets, a total of 85 species were identified. Nudibranch populations showed high levels of diversity within an uneven, unstable community. Citizen scientist data provided imperative information to the baseline assessment and, therefore, the inclusion of these data increased the robustness of this study. Environmental and experimental variables investigated did not influence the outcomes of this study and should therefore not be heavily focused on in designing future experiments. Future monitoring studies should continue to record oceanic pH in order to detect any possible changes due to ocean acidification. It is recommended that sampling events should be increased in order to capture all species present in these localities. These events should also encompass an extended temporal scale in order to cover a larger temperature range. Research on bioindicators is essential within today's rapidly changing climate, mainly due to human activities, particularly within an extremely vulnerable habitats such as coral reefs.

## Graphical abstract

The study emphasises the importance of monitoring nudibranch populations as bioindicator species within marine ecosystems, as they can provide valuable information on fine‐scale environmental changes influenced by climate change, highlighting the need to establish baseline analyses and investigate environmental and experimental parameters for future sampling procedures. To effectively detect changes caused by ocean acidification, future monitoring studies should regularly measure oceanic pH, increase sampling events to encompass all species present, extend the temporal scale to cover a wider temperature range, and prioritise research on bioindicators, especially in vulnerable habitats like coral reefs, given the rapid climate change caused by human activities.

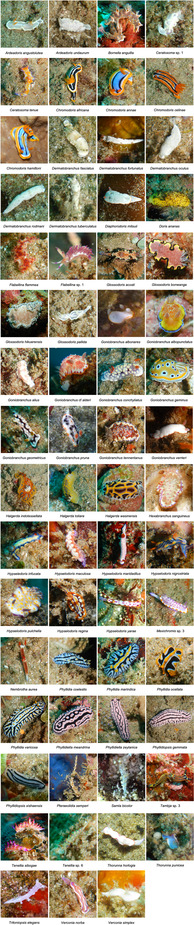

## 1 INTRODUCTION

Resource availability of all ecological communities is chronically altered by drivers of global change (Koerner et al., 2015). Resources such as nitrogen deposition, deviations in precipitation, and increasing carbon dioxide levels are being impacted (Koerner et al., 2015). Anthropogenic activities are accelerating the rates of global change at an alarming speed by intensifying the effect of these drivers (Ingole, 2021). It is anticipated that these alterations in resources will have large impacts on ecosystem functioning through transforming community structure and composition (Koerner et al., 2015). Marine ecosystems form intricate food webs and are thus characterised by complex biological interactions that directly or indirectly impact the success of species through the performance of another (Doney et al., 2012). The main physiological effects that rising atmospheric carbon dioxide levels and global climate change will have on marine environments will include concurrent increases in temperature, changes in circulation, increased stratification, changes in nutrient input, ocean acidification, and decreased oxygen content (Brierley & Kingsford, 2009; Doney et al., 2012). These changes are expected to affect the natural composition of marine populations by causing phenological disruptions, shifts in abundance, as well as changing the spatial organisation of organisms such as distribution and dispersal (Brierley & Kingsford, 2009; Doney et al., 2012). An effective method for assessing the effects that ecosystems experience due to climate change can be achieved through the use of bioindicator species (Cooper et al., 2009).

Bioindicators are organisms that reflect the health or quality of an ecosystem (Jackson et al., 2000; McGeoch, 1998; Siddig et al., 2016). They are therefore frequently used by natural resource managers for assessing the well‐being of ecosystems, as well as providing early warning signs of environmental deterioration (Jackson et al., 2000; McGeoch, 1998; Siddig et al., 2016). The presence of bioindicator species can convey important information about biological, chemical, or physical changes occurring in the environment and can therefore indicate major stressors experienced by ecosystems (Niemi et al., 2004; Niemi & McDonald, 2004). The use of bioindicators is becoming increasingly prevalent with natural resource management agencies as they provide an easy, rapid, and inexpensive method in depicting environmental degradation on a larger scale (Cooper et al., 2009; Navarro‐Barranco et al., 2020). It is anticipated that marine bioindicator organisms will be crucial in the conservation and management of coral reefs worldwide by highlighting changes within the physical environment (Dale & Beyeler, 2001; Navarro‐Barranco et al., 2020; Niemi et al., 2004). Due to their unique life history traits and ability to indicate change, this study sets out to suggest the use of nudibranchs as essential bioindicator organisms to understand the effects of climate change on coral reef ecosystems (Goddard et al., 2011; Nimbs et al., 2015).

Nudibranchs are a diverse group of brightly coloured marine gastropod molluscs that shed their shells in the larval stage of development and thereafter remain shell‐less (Dean & Princep, 2017; Dumdei et al., 1989). There are over 4700 known species of nudibranchs (Gofas, 2021) which inhabit a diverse array of habitats. These habitats include both tropical and subtropical marine environments (Clark, 1975; Debelius, 1996; Johnson & Gosliner, 2012), with some species being seen to inhabit brackish‐waters (Korshunova et al., 2018; Swennen, 1996).

As they are essentially blind, nudibranchs perceive their environment through two specialised rhinophores that are chemosensory structures (Arey, 1918; Murphy & Hadfield, 1997). The rhinophores are used to interpret chemical cues transported by water currents in order to locate food or potential mates (Arey, 1918; Wertz et al., 2006). Due to their reliance on chemoreception, nudibranchs are sensitive to changes in ocean water parameters that are affected by global warming, such as changing pH associated with ocean acidification (Albright et al., 2018; Kurnianda et al., 2020; Seroy & Grünbaum, 2018). Nudibranchs are also susceptible to environmental changes that affect the substrate on which they depend on for feeding and reproduction (Biermann et al., 1992; Burn, 2015; Johnson & Gosliner, 2012). Species in this order are carnivorous benthic organisms that prey on coral, sponges, hydroids, sea anemones, fish eggs, barnacles, as well as other nudibranchs that they can locate on the sea floor (Kara et al., 2018). With only a few known exceptions, nudibranchs are generally seen to be hermaphrodites (Burn, 2015; Sekizawa et al., 2013), with both male and female apertures located within a common chamber called an atrium on the anterior right side of their bodies (Burn, 2015). During mating, individuals align so that there is contact between their reproductive apertures (Burn, 2015). Nudibranchs then deposit spirally coiled gelatinous egg ribbons onto hard surfaces (Biermann et al., 1992; Burn, 2015). The adult nudibranchs die once the egg‐laying process is complete; thus, the life‐cycle is short‐lived (Burn, 2015; Costello, 1938). Smaller species have a life span of 4–6 weeks, whereas some larger species may live approximately 2–3 years (Burn, 2015). Their short life span allows them to respond rapidly to changing environmental conditions (Kurnianda et al., 2020). Overall, due to their life history traits, reliance on chemoreception, and their ability to rapidly adapt, nudibranchs make for key bioindicator species of reef health, as well as environmental changes (Goddard et al., 2011; Nimbs et al., 2015). These traits make long‐term data on diversity, abundance, and biogeography of nudibranchs essential for understanding the effects of climate change on marine ecosystems.

Many studies to date have been conducted almost entirely on the bioactive metabolites and taxonomy of nudibranchs, whereas only a few studies have focused on diversity and abundance (Sabdono et al., 2021). None of these limited studies have been conducted in Sodwana Bay, and therefore, this study is the first of its kind. The importance of this study is that it lays the foundation for future studies to investigate nudibranchs as bioindicators of climate change, as well as the effect that climate change will have on the coral reefs of Sodwana Bay.

In order to establish nudibranchs as bioindicators in future research, the main aim of this study was to provide a baseline assessment of nudibranch diversity on Two‐Mile Reef, a marine protected area, located in Sodwana Bay, South Africa. This was assessed through an underwater visual census of nudibranchs at three dive sites. Citizen scientist data of nudibranch observations were analysed in parallel with scientific findings in order to increase the robustness of the census. Additionally, the influence of environmental and experimental parameters on nudibranch observations was investigated. This was done to determine if sampling procedures are affected by these factors. Finally, the results of this study will be used to recommend the most efficient sampling methods to assess nudibranch diversity for long‐term monitoring programs. Scientific findings will be logged into a sample‐based (SB) dataset and citizen scientist data into a separate incidence‐based (IB) dataset. Within the SB dataset, it is predicted that diversity among the three study sites will be similar, as indicated by the Shannon–Weaver index. It is also anticipated that the uniformity index will show that an unstable community is present at all three sites, due to differences in abundance among species. *Chromodoris hamiltoni* Rudman, 1977, and *Chromodoris celinae* Tibiriçá, 2019, are foreseen to be the most common species at all the sites and will therefore affect the Simpson Dominance index. Other species expected to be observed include *Chromodoris africana* Eliot, 1904, *Chromodoris quadricolour* Ruppell & Leuckart, 1828, *Glossodoris bonwanga* Matsuda & Gosliner, 2018, *Halgerda wasinensis* Eliot, 1904, and *Phyllidiella zeylanica* Kelaart, 1859.

## 2 MATERIALS AND METHODS

### 2.1 Location and timing

**FIGURE 1:**
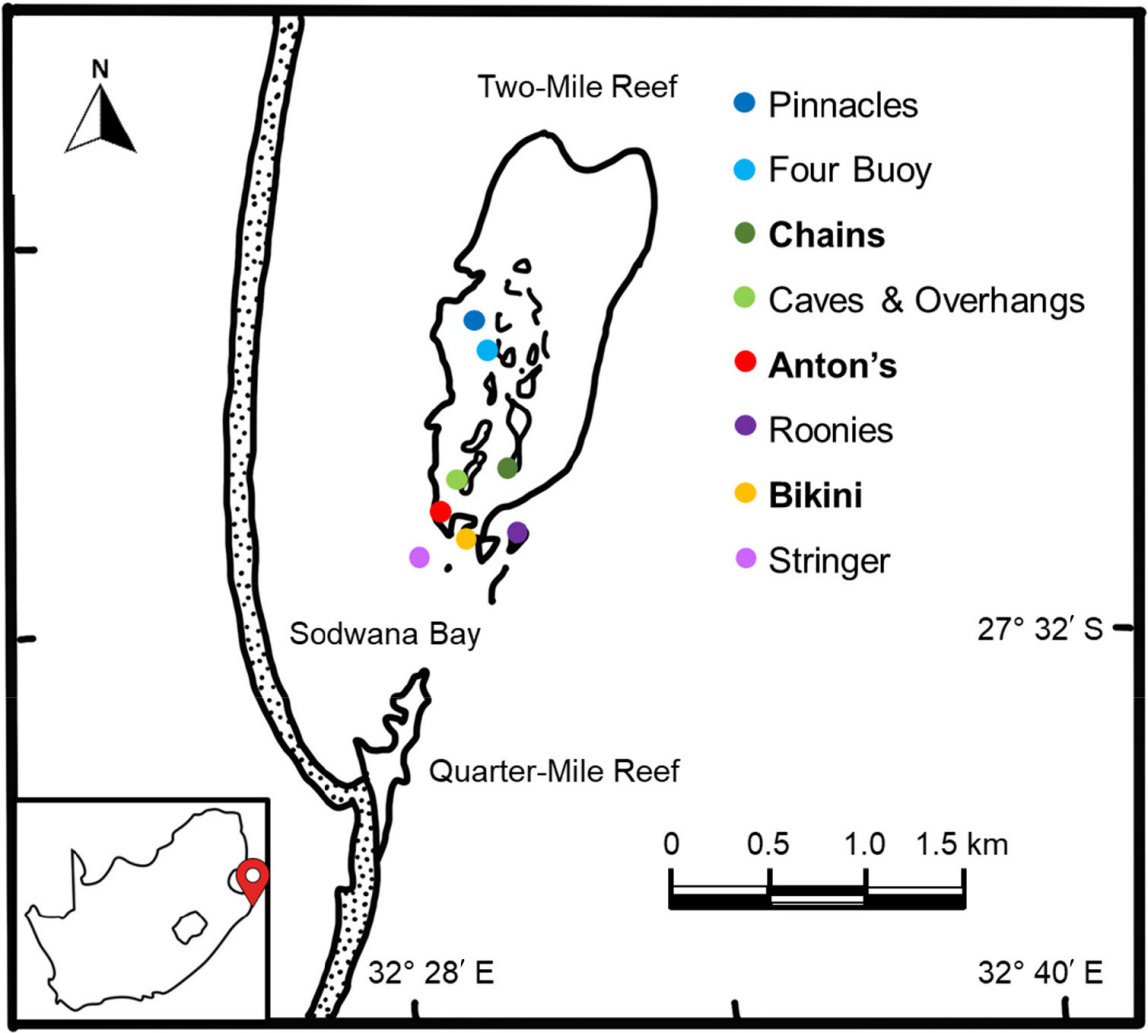
A map of Two‐Mile Reef off Sodwana Bay, South Africa. The three study sites where nudibranch sample‐based data were collected are Chains, Anton's, and Bikini (indicated in bold on the top right‐hand corner). The dive sites where incidence‐based observations were made are also listed on the top right‐hand corner. Coloured circles on the map represent the dive sites listed.

Sample‐based (SB) observation data were collected in the marine protected area of Sodwana Bay, South Africa across three different dive sites. All three of the study sites are located on Two‐Mile Reef. These dive sites were Chains, Anton's, and Bikini reef with the maximum depth for each study site at 17.00, 15.00, and 21.00 m, respectively (Figure 1). Data collection was conducted over a period of 2 weeks during April and May 2022. Incidence‐based (IB) observations from citizen scientists were collected across 8 dive sites on Two‐Mile Reef and included the three sites for the SB observations (Figure 1, Appendix 1). The depth for the IB observations ranged from 11.80 to 32.60 m. Incidence‐based observations from citizen scientists were collected from February to July 2022.

### 2.2 Sample‐based data collection

**FIGURE 2:**
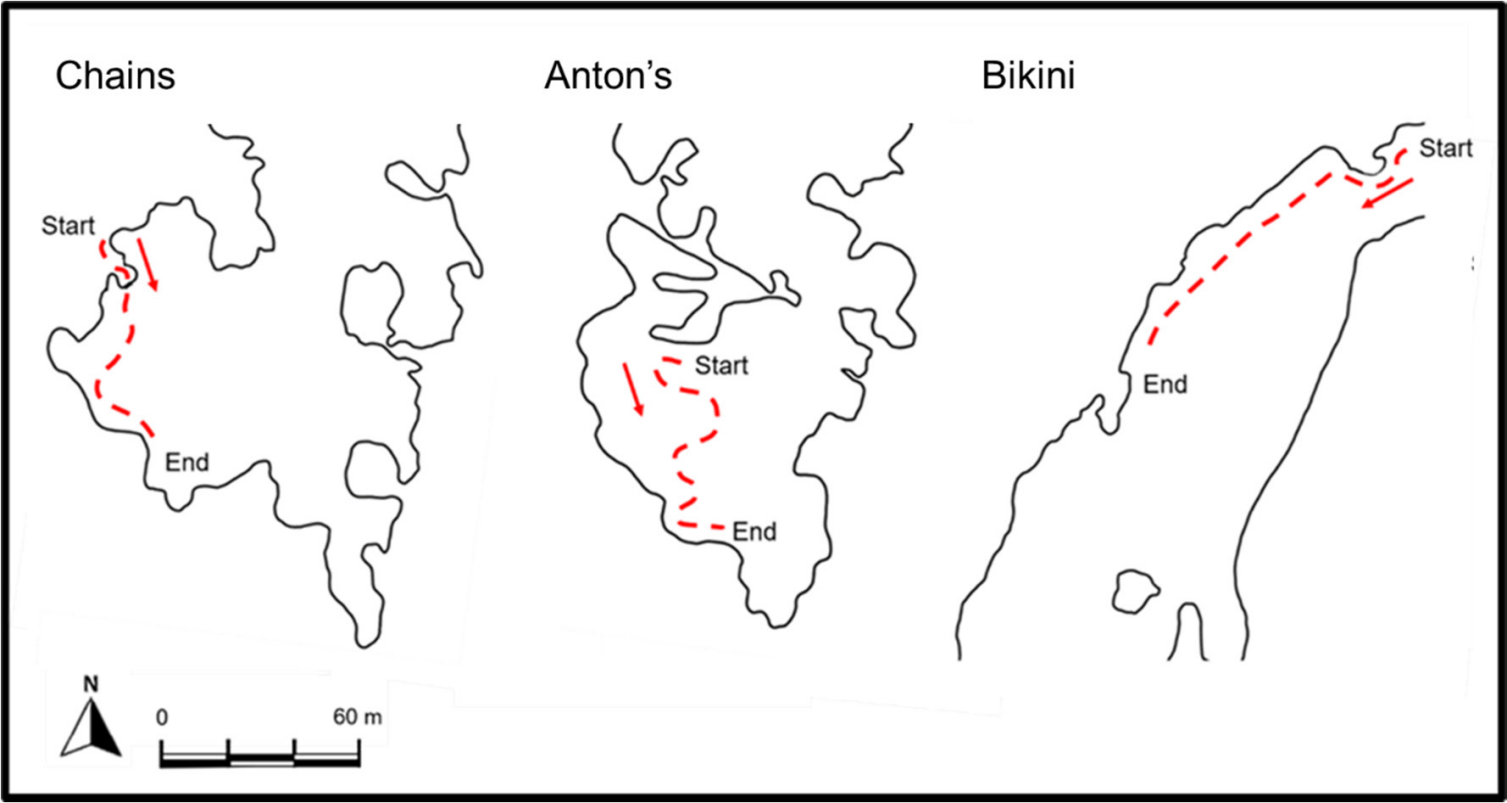
A map of each dive site for the sample‐based dataset; Chains, Anton's, and Bikini Reef. The red broken line indicates the route that was followed each time the dive site was sampled. The start and end point are depicted on the route as well as an arrow to indicate the direction of travel.

Underwater visual census were utilised to obtain data on nudibranchs. This involved a team of SCUBA divers observing nudibranchs along a designated route set out across each dive site (Figure 2). Each study site was sampled five times over the period of 2 weeks. Each nudibranch observed was photographed using a Nikon Coolpix w300 underwater camera. Species were then identified using Nudibranch & Sea Slug Identification – Indo‐Pacific 2nd Edition (Gosliner et al., 2015), A Guide to the Sea Slugs of the Maputaland Coast (Strö̈mvoll & Jones, 2019), The Reef Guide (King, 2014), The Sea Slug Forum online database (Australian Museum, 2010), and the Nudibranch ID Indo Pacific application (Cobb, 2022). Journal articles were also utilised for identifying species within the *Chromodoris* (Tibiriçá et al., 2019) and *Flabellina* (Ekimova et al., 2022) genera. Species were logged into a dataset for each study site. Various parameter measurements were collected in situ for each of the dive sites using a Thermo Fisher Scientific handheld conductivity metre and a Suunto Zoop dive computer. Environmental parameters collected included temperature (°C) and acidity (pH). A purposive sampling method was used to obtain water parameters, such as visibility, and the strength of the surge and current. Experimental parameters such as dive time, maximum depth, visibility, and number of observers were also recorded for each sampling event.

### 2.3 Incidence‐based data collection

Observations from citizen scientists in Sodwana Bay were obtained through the Deep Blue Conservation organisation. Nudibranchs were also photographed with a Nikon Coolpix w300 underwater camera. Identification of species were carried out using the same resources as utilised in the SB dataset. Environmental parameters collected by citizen scientists included temperature (°C), strength of the surge, and the strength of the current experienced during each dive. Experimental parameters collected included dive time, maximum depth, visibility, and number of observers.

### 2.4 Data analysis

#### 2.4.1 R analysis

Due to the different sampling methods employed for the SB data and IB data, they were treated as separate datasets in all the analyses. R version 4.1.2 (R Core Team, 2021) was used for all statistical analyses in this study. Generalised linear models were used to test for significant differences between species richness and abundance between sample sites in the SB dataset. A Venn diagram was produced to show common or unique species belonging to the two datasets. Species accumulation curves were generated for each dataset. Sample‐based data were used to create a species accumulation curve based on sampling events. The number of individuals was used to produce the species accumulation curve based on IB data. A rank abundance curve of SB data was produced for all species observed across the three dive sites. A second rank abundance curve of IB data was also produced. The Shannon–Weaver index as well as the Simpson dominance index were calculated in RStudio using the “diversity ()” function. These values were obtained for each sampling event on each of the dive sites and an average was then obtained for the SB dataset only. The uniformity index values were calculated manually with the formula below using the average Shannon–Weaver index for each dive site. Generalised linear models were constructed using the environmental and experimental parameters as predictor variables, with nudibranch abundance or species richness as the response variable. An ANOVA was then performed to test for a significant effect of the predictor variables on the response. Significance of both environmental and experimental parameters on species composition was also obtained using a permanova test.

### 2.5 Diversity indices

#### 2.5.1 Shannon–Weaver index

The Shannon–Weaver index (*H′*) was used as an index of diversity in this study. The *H′* was calculated for each sample site in order to provide a baseline diversity level for Chains, Anton's, and Bikini in the SB dataset (Clarke & Warwick, 2001). The formula used was (Shannon & Weaver, 1998):

$$H^{\prime }=-\sum_{i=1}^{S}\mathrm{Pi}\ln\mathrm{Pi}.$$

where *Pi* = *ni*/*N*, with *ni* as the number of individuals of each species (*i*th species) with *N* as the total number of individuals in the sample (Paulangan et al., 2021). The results obtained for the Shannon–Weaver index was compared to the criteria for assessing the results (Table 1).

**TABLE 1:** The criteria for interpreting the Shannon–Weaver index (*H′*) of diversity utilised in this study (Paulangan et al., 2021; Shannon & Weaver, 1998).

Shannon–Weaver index (*H′*) values	Diversity ranking
*H′* > 3.0	Very high
1.6 < *H′* < 3.0	High
1.0 < *H′* < 1.5	Moderate
*H′* < 1.0	Low

#### 2.5.2 Uniformity index

The uniformity index (*E*) describes the number of individuals between species within a community (Ulfah et al., 2019). If the individuals between species are evenly distributed then the more balanced the ecosystem will be (Ulfah et al., 2019). The formula used is (Odum, 1967):

$$E=\frac{H^{\prime }}{H_{\max}}$$

where *H*′ is the Shannon–Weaver index and *H*_max_ = ln(*S*), where *S* is the total number of species found (Krebs, 1999). The uniformity index falls within a range 0–1 and follows the proceeding criteria (Table 2).

**TABLE 2:** The criteria for interpreting the uniformity index (*E*) of community stability utilised in this study (Krebs, 1999).

Uniformity index (*E*) values	Stability of community
0.00 < *E* ≤ 0.50	Depressed
0.50 < *E* ≤ 0.75	Unstable
0.75 < *E* ≤ 1.00	Stable

A low uniformity index shows an uneven distribution of species within the community where one species tends to dominate (Ulfah et al., 2019). A high uniformity index shows evenness between the number of individuals of each species that inhabit a community (Ulfah et al., 2019).

#### 2.5.3 Simpson dominance index

The Simpson dominance index (*C*) was used to assess the evenness of species composition within a community (Paulangan et al., 2021). The formula used (Odum, 1967) is as follows:

$$C=\sum_{i=1}^{s}{\mathrm{Pi}}^{2}$$

where *Pi* = *ni*/*N*, with *ni* as the number of individuals of each species (*i*th species) and *N* as the total number of individuals in the sample (Odum, 1967). Much like the uniformity index, the dominance values also range between 0–1 and are assessed according to the following criteria (Table 3).

**TABLE 3:** The criteria for interpreting the Simpson Dominance index (*C*) of species evenness within a community utilised in this study (Odum, 1967).

Simpson dominance index (*C*) values	Evenness
0.00 < *C* < 0.50	Low
0.50 < *C* ≤ 0.75	Moderate
0.75 < *C* ≤ 1.00	High

## 3 RESULTS

A total of 290 nudibranch individuals were successfully photographed and identified in the SB dataset. In total, 63 species belonging to 13 different families were observed across all three sites. The most predominant family observed was Chromodorididae with 35 out of 63 species (55.56%) belonging to this family. *Halgerda wasinensis* Eliot, 1904 (Appendix 2) was the most abundant species with a total proportion of 20%. The second most abundant species was *G. bonwanga* that occurred at a proportion of 16.55% (Appendix 2). The total number of species observed at each dive site was 29 species at Chains, 31 at Anton's, and 32 at Bikini. Some species were unique to a particular dive site (Appendix 3). The total number of species unique to Chains was 11, 13 to Anton's, and 16 to Bikini. These unique species along with all other species detected in the SB dataset are viewed in Appendices 2 and 3. Neither the number of species (*p* =.65) nor species abundance (*p* =.29) at the three dive sites were significantly different from each other.

**FIGURE 3:**
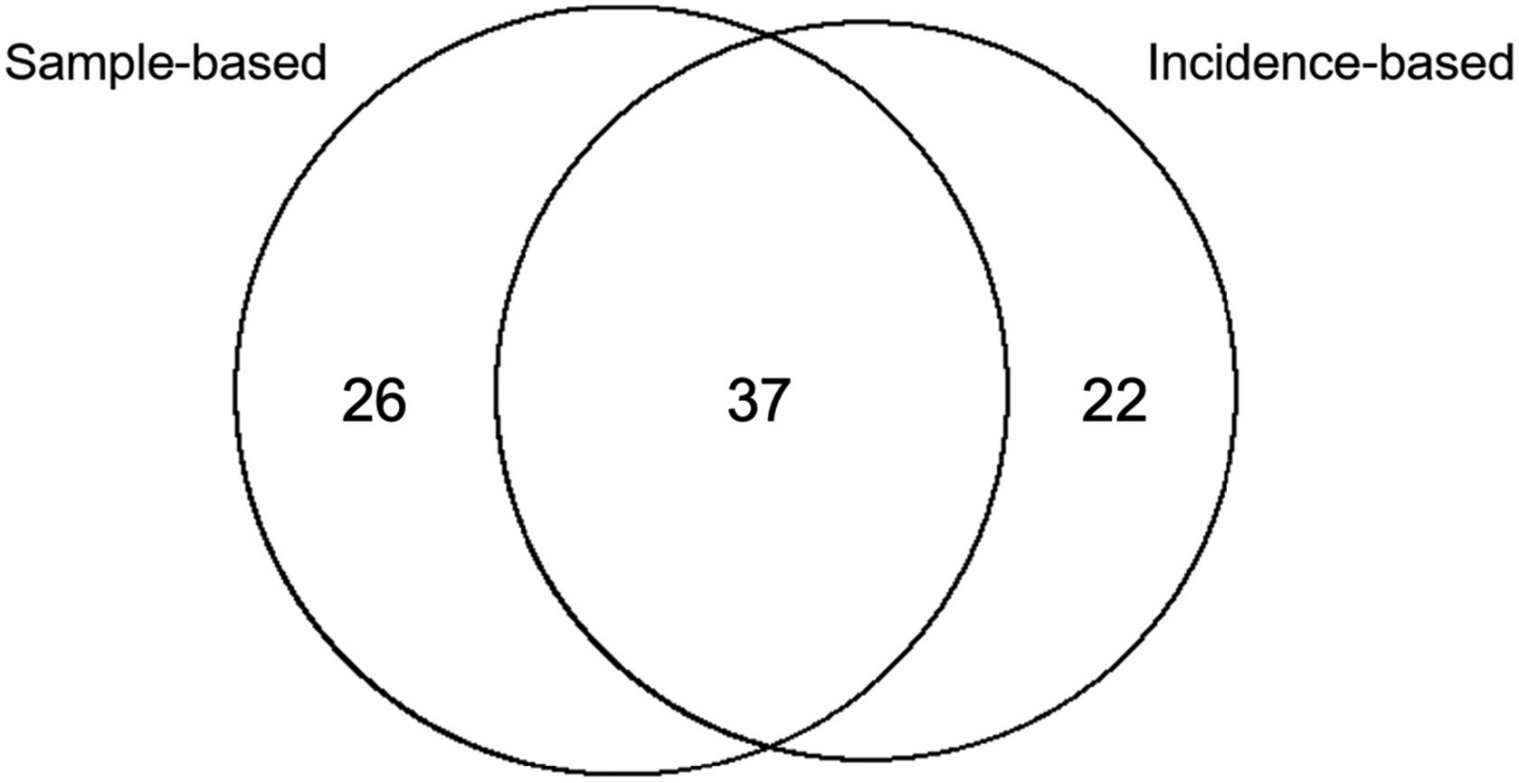
A Venn diagram depicting the number of species shared by the two datasets as well as the number of species unique to the sample‐based and incidence‐based datasets.

Within the IB dataset, a total of 58 species were identified that belong to 14 different families. These species were identified from photographs taken of 342 nudibranch individuals on Two‐Mile Reef across eight dive sites. Yet again, Chromodorididae was the most proliferate family as it was represented by 23 out of the 58 species (38.33%). The most abundant species was once again *H. wasinensis* with 17.84% of the total individuals observed. *Chromodoris hamiltoni* Rudman, 1977 (Appendix 2) was the second most abundant species with a proportion of 17.24% of the individuals. All species contained in the IB dataset, along with the respective dive sites that they were observed at, are viewed in Appendix 4. The two datasets shared 37 species with 26 species unique to the SB dataset and 22 to the IB dataset, bringing the total identified species to 85 (Figure 3).

**FIGURE 4:**
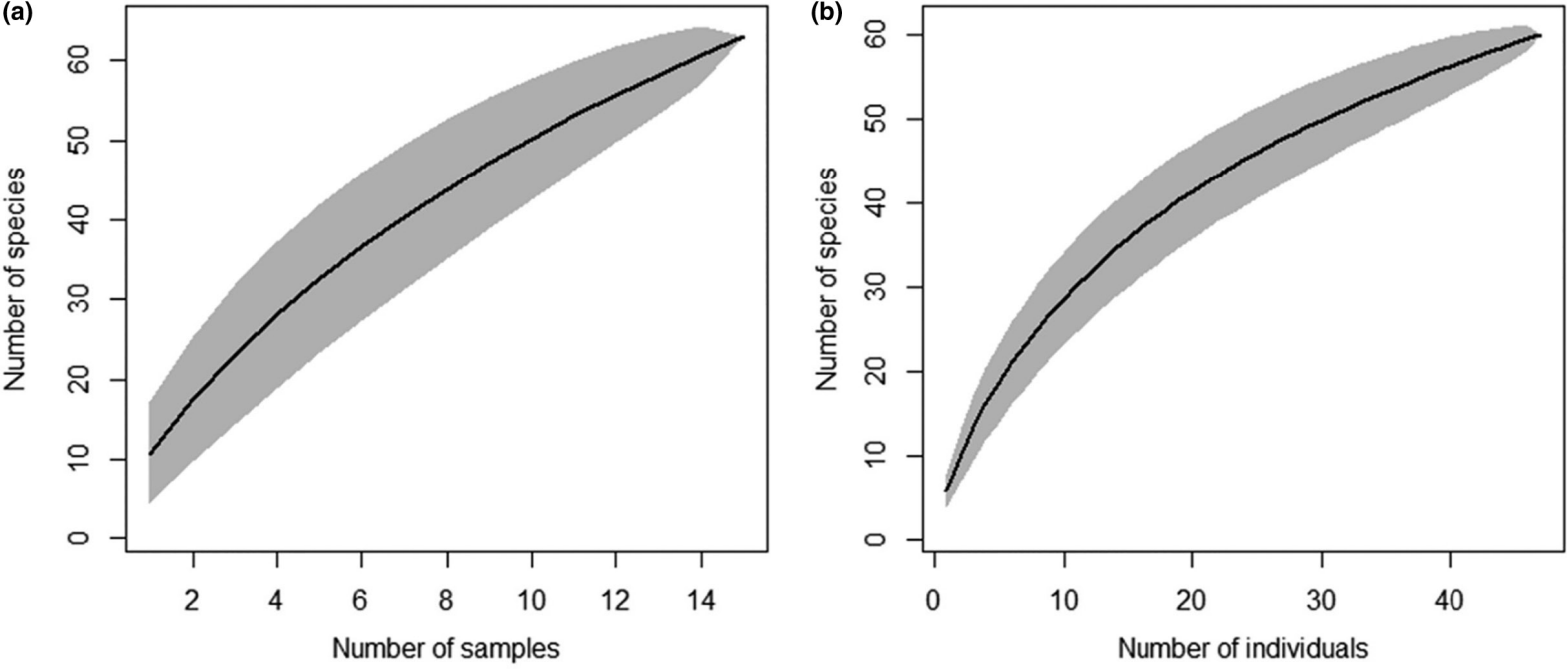
Species accumulation curves with (a) generated from the sample‐based dataset and (b) generated from the incidence‐based dataset.

The species accumulation curve generated from the SB dataset (Figure 4a) showed incomplete sampling. An asymptote has not been reached within this dataset showing that not all species that are present were sampled. This curve indicates that the total species observed would increase until reaching an asymptote if additional sampling events were undertaken. Similarly, the species accumulation curve of the IB dataset also depicts incomplete sampling (Figure 4b). However, the curve is closer to reaching an asymptote when compared to that of the SB dataset (Figure 4a).

**FIGURE 5:**
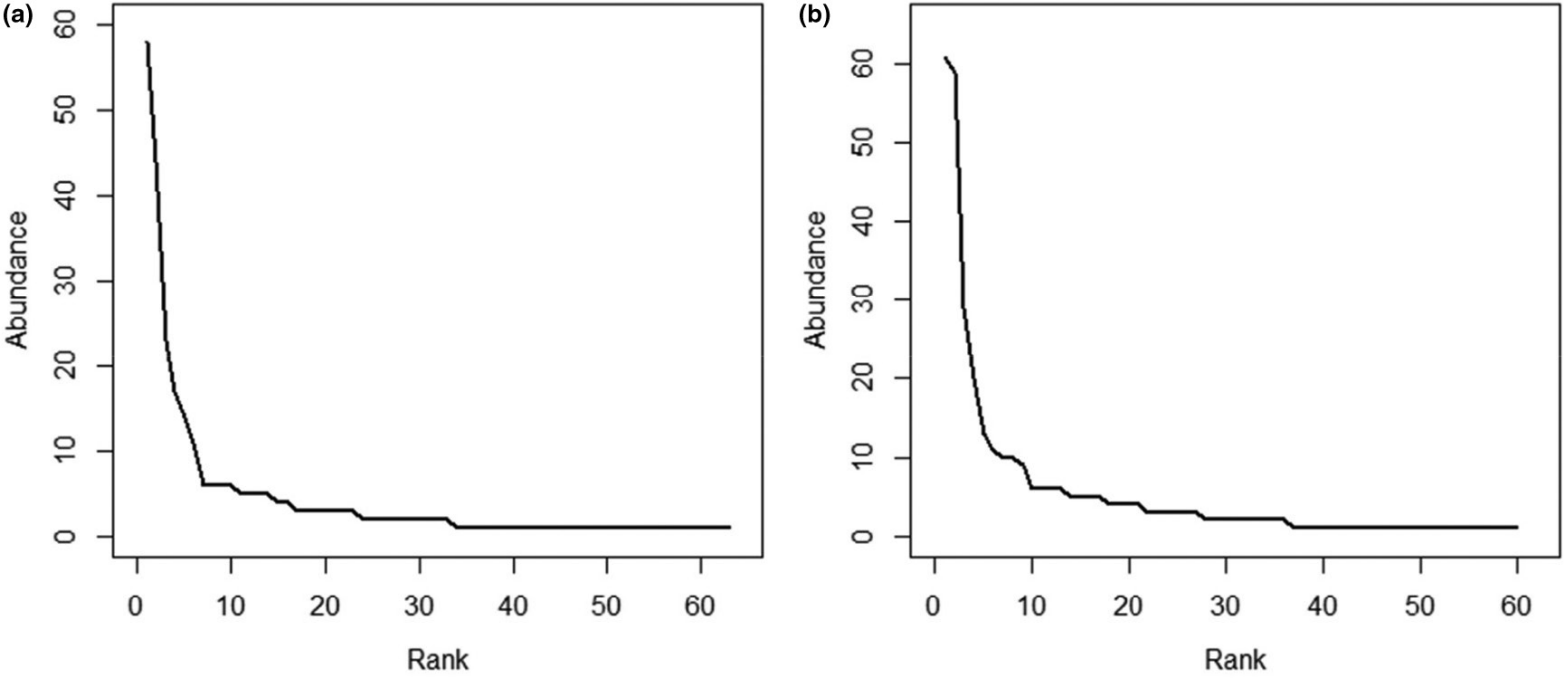
Rank abundance curves with (a) generated from the sample‐based dataset and (b) generated from the incidence‐based dataset.

The rank abundance curve produced from the SB dataset (Figure 5a) indicates that there are levels of unevenness in the community. Higher ranked species occur at greater abundances and therefore dominate. There is a large disparity between the abundance of the highest ranked species and those that proceed them. The lowest ranked species occur at similar abundances as the curve remains approximately horizontal as rank decreases. A very similar rank abundance curve was generated from the IB dataset (Figure 5b). This curve also depicts unevenness within the community and a large disproportion in abundance between higher and lower ranked species.

The diversity indices were calculated for the SB dataset only. The Shannon–Weaver index ranged between 1.56 and 2.80 across all three dive sites. The average Shannon–Weaver index for Chains, Anton's, and Bikini was calculated to be 2.13, 2.12, and 2.23 respectively. The overall average for the Shannon–Weaver index for all three dive sites was 2.16. The Simpson dominance index ranged between 0.77 and 0.93 across all three dive sites. The average Simpson dominance index for each of the dive sites was determined to be 0.84, 0.86, and 0.87 for Chains, Anton's, and Bikini, respectively. The overall average Simpson dominance index for all three sites was 0.86. The average uniformity index for each dive site respectively was 0.64, 0.62, and 0.63 for Chains, Anton's, and Bikini. The average uniformity index across all three dive sites was 0.63. All data regarding the diversity indices are viewed in Appendix 5.

The pH ranged from 7.96–8.17 across all three dive sites. The overall average pH was recorded to be 8.07. The surge was never strong and varied between none, low, and medium. The current was only seen to be slight or no current present. Maximum depth was recorded to be within the range of 22.60–15.40 m with an average maximum depth of 18.52 m. The longest dive time was recorded to be 75.00 min compared to the shortest dive time of 42.00 min. The average dive time spent across all dive sites was 58.13 min. The water temperature was not tested for significance on nudibranch assemblages since it was constant at 24°C throughout the study except on the fifth dive on Bikini when it decreased by 1°C. This trend was also seen to be true for visibility which remained constant at approximately 20.00 m. The generalised linear model testing for a significant effect of environmental and experimental parameters on nudibranch abundance, showed that none of the predictor variables significantly affected the response (*p* > .05) (Table 4). The second generalised linear model testing for a significant effect of environmental and experimental parameters on nudibranch species richness, again displayed that none of the predictor variables significantly affected the response (*p* > .05) (Table 5). The permanova test showed that no water or dive parameters had a significant effect on nudibranch composition (*p* > .05) (Table 6). Overall, none of the water or dive parameters assessed in this study significantly affected the nudibranch assemblages sampled.

**TABLE 4:** A summary of the generalised linear model investigating the effect of environmental and experimental parameters (predictor variables) on nudibranch abundance (response variable).

Water parameters	LR chi‐sq	df	*p*‐Value
pH	1.368	1	.242
Strength of the current	0.635	1	.426
Strength of the surge	0.495	2	.781
Dive time	0.552	1	.457
Maximum depth	0.281	1	.596
Number of observers	0.025	1	.874

**TABLE 5:** A summary of the generalised linear model investigating the effect of environmental and experimental parameters (predictor variables) on species richness (response variable).

Water parameters	LR chi‐sq	df	*p*‐Value
pH	0.863	1	.353
Strength of the current	1.250	1	.264
Strength of the surge	0.006	2	.997
Dive time	1.691	1	.193
Maximum depth	1.185	1	.276
Number of observers	0.791	1	.374

**TABLE 6:** A summary of the permanova test investigating the effect of environmental and experimental parameters (predictor variables) on species composition (response variable).

Water parameters	*R* ^2^	*df*	*p*‐Value
pH	.072	1	.391
Strength of the current	.076	1	.294
Strength of the surge	.128	2	.483
Dive time	.094	1	.135
Maximum depth	.072	1	.383
Number of observers	.071	1	.402

## 4 DISCUSSION

This study set out to quantify nudibranch communities within Sodwana Bay, South Africa, to provide a baseline assessment for future studies investigating the use of nudibranchs as bioindicators of climate change. Since this was the first study of its kind, another important aim was to investigate the environmental and experimental variables that may influence sampling. This investigation could then be used to make recommendations for improved experimental design. Nudibranch communities studied within the SB dataset indicate high levels of diversity, as shown with the combined average Shannon–Weaver index value (Appendix 5). Dominance of species is portrayed by the Simpson dominance index which indicates that there is an uneven distribution of species (Appendix 5). The species that dominated within the SB dataset were *H. wasinensis* and *G. bonwanga*. However, in addition to *H. wasinensis*, *C. hamiltoni* was highly predominant in the IB dataset. This uneven spread of species is further depicted by the rank abundance curves (Figure 5). These curves show the disparity in abundances of species at higher and lower ranks. Uneven distribution of species, with levels of dominance, is further shown using the uniformity index. This index depicts an unstable community which cooperates with conclusions drawn from the Simpson dominance index and rank abundance curves (Figure 5, Appendix 5).

Corresponding results from a study conducted in southern Mozambique, 200 km north of Sodwana Bay, found *H. wasinensis* to be the most common species (Tibiricá et al., 2018). *Chromodoris hamiltoni* is also known to be a proliferate species found along subtidal reefs located on the east coast of Africa (McPhail & Davies‐Coleman, 1997). On the other hand, *G. bonwanga* is a newly described species (Matsuda & Gosliner, 2018). Thus, there is no data available regarding the abundance or possible reasons for the dominance of this species along the southern African coastline. Therefore, it is important that aspects like breeding patterns and diet be thoroughly investigated in future studies in order to address this gap in our understanding of these observations. The diversity indices can be utilised as baseline values for future studies to track nudibranch community level changes. The method of repeated sampling along designated routes was seen to be effective in quantifying nudibranch communities. The success of this was shown through no significant difference between nudibranch abundance or species richness between sample sites.

The average pH recorded was 8.07 which is in line with the current global average of 8.10 (Fassbender et al., 2021). This recorded value can be used to detect minor changes in pH within the Sodwana Bay marine protected area. Oceanic pH levels are expected to decrease in the near future due to the increasing levels of atmospheric carbon dioxide, resulting in an increased level of absorption by the ocean (Feely et al., 2009; Orr et al., 2005). Preliminary readings, as done here, become extremely important in monitoring the effects of ocean acidification on marine ecosystems.

Research has shown the potential of nudibranchs to act as bioindicators of climate change, as well as reef health. This is due to the influence of climate change on nudibranch abundance through the alteration of environmental variables. Total nudibranch abundance is highly correlated with ocean water temperature (Goddard et al., 2011, 2013; Schultz et al., 2011; Wilson et al., 2016) which is shown through population fluctuations in accordance to El Niño and La Niña events (Goddard et al., 2011). During periods of warming, such as those associated with El Niño events and the warm phase of the Pacific Decadal Oscillation, there is a strong positive correlation in nudibranch abundance (Goddard et al., 2011). These climatic events strongly impact sea surface temperature as well as sea surface height, which ultimately effects abundance (Goddard et al., 2011). Contrary to this, abundance is negatively correlated to periods of cooling, such as La Niña events, the cold phase of the Pacific Decadal Oscillation, heightened coastal upwelling, and the positive phase of the North Pacific Gyre Oscillation (Goddard et al., 2011). This dependency on ocean water temperature is precipitating range shifts along with regional declines of certain nudibranch species due to climate change resulting in warming sea temperatures (Goddard et al., 2011; Nimbs et al., 2015). Range shifts such as those investigated by Goddard et al. in 2011 can result in ecological disruption and thus the importance of understanding how nudibranchs are influenced by environmental factors in order to predict future potential impacts on ecosystem functioning.

Potential draw backs of this study can be attributed to the cryptic nature of nudibranch species (Churchill et al., 2014; Epstein et al., 2019; Fritts‐Penniman et al., 2019; Matsuda & Gosliner, 2018). Due to this, there is potential for cryptic species to be nested within those known to science. Without taxonomic evaluation through genetic assessment or analysis of radular morphology and reproductive systems, the identification of these cryptic species is highly unlikely. An example of this can be seen in juveniles of certain species of the *Chromodoris* genus, which are very difficult to distinguish from each other. During early life stages these species do not possess the distinguishing characteristics that the adult individuals show. This was a challenge encountered by citizen scientists that were unable to identify to which species a *Chromodoris* juvenile should be assigned to. Citizen scientists were also unable to identify species belonging to the *Dermatobranchus* genus as it is dominated by cryptic species that have similar external anatomy, and can predominantly be distinguished using internal anatomy (Gosliner & Fahey, 2011). With this being said, nudibranch individuals in the SB dataset were identified through a rigorous process using numerous resources such as, identification guides, online databases, applications, expert consultation, and published journal articles, in order to increase the likelihood of correct classification.

Environmental and experimental factors in this study were not seen to influence nudibranch abundance, species richness, or composition (Tables 4, 5, 6). These factors included strength of the surge and current, depth, dive time, or number of observers. It can therefore be suggested that these variables should not influence future sampling procedures and do not need to be factored into experimental designs. However, there were methodical limitations present in this study that can be used to enhance future census. Firstly, sampling occasions within the SB dataset were not sufficient to accurately quantify nudibranch populations (Figure 4). Consequently, increasing these events is strongly advised in order to sample all species present. Secondly, temperature remained constant during the sampling period and could not be incorporated into the results. It is suggested that multiple sampling occasions should be completed throughout the year in order to obtain an adequate temperature range. This extension of sampling procedures could also be beneficial as it could encompass more variation within surge and current that was not obtained here. Through the use of photography, distinguishing morphological characteristics could be identified for accurate classification of species that would otherwise be difficult to recall from memory. Photographing individuals is a useful tool and should therefore be continued in future studies.

Data gathered by citizen science has extensively been used by scientists, policy makers, and the general public (Miller‐Rushing et al., 2012; Silvertown, 2009). With the aid of citizen scientists, ecological research data can be collected at unprecedented spatial and temporal scales (Dickinson et al., 2012). This is possible through the assembly of historical data, and a large, distributed team of knowledgeable observers (Dickinson et al., 2012). Gathered data can then be employed in critical baseline studies to either respond to crises or identify threats to wildlife and/or people (Dickinson et al., 2012). Along with this, citizen science encourages the public to become involved in scientific research (Kobori et al., 2016). By integrating the public into such research, education on important matters such as climate change and environmental stewardship is enhanced (Marshall et al., 2012). This study is evident of the important contributions of citizen scientists. A total of 22 new species were added to the baseline assessment through the incorporation of the IB dataset. Inclusion of this dataset also enabled the investigation of a broader temperature range on nudibranch seasonality. Additionally, volunteers forming part of Deep Blue Conservation were exposed to important concepts such as climate change and using bioindicators to monitor variation in environmental conditions. The incorporation of citizen scientists has been beneficial in this study and should continue to be made use of in future research.

Studies similar to this one are extremely important for laying down a foundation for building, and extending monitoring programs in the future. Within marine environments, these baseline studies are imperative due to degradation being more pervasive than land (Knowlton & Jackson, 2008). Baseline studies are particularly scarce for marine benthic ecosystems such as coral reefs (Knowlton & Jackson, 2008). This lack of information is alarming as these ecosystems are among the most highly threatened by global climate change (Bellwood et al., 2004; Hoegh‐Guldberg et al., 2007). To date, many of the ecological studies of coral reefs have been conducted on systems that are already heavily degraded by human impacts (Knowlton & Jackson, 2008). These studies do not provide accurate baseline assessments as they quantify already degraded environments and are therefore not a precise representation of ecosystem functioning (Knowlton & Jackson, 2008). Since Sodwana Bay was declared a protected area in the 1950s, it is a pristine coral reef system (Schleyer & Tomalin, 2000) on which an accurate baseline assessment can be conducted. With nudibranchs at its centre, this study can therefore provide an explicit baseline analysis that can be used to precisely quantify future impacts of climate change on this ecosystem. Although nudibranchs do not possess all the typical characteristics of bioindicator organisms such as well‐studied taxonomy and ecology (Holt & Miller, 2011), their life history traits, reliance on chemoreception, and their ability to rapidly adapt deem them worthwhile to justify future research to investigate their bioindicator potential.

## 5 CONCLUSION

Nudibranch populations on Two‐Mile Reef can be characterised as having a high diversity, uneven distribution of species, and unstable composition. A total of 85 species were identified in both the SB and IB datasets combined. Dominant species observed in the two datasets included *H. wasinensis*, *G. bonwanga*, and *C. hamiltoni*, which corresponds with other research indicating that these species are common along the east coast of Africa. However, there is a data gap for *G. bonwanga* as it is a newly described species and therefore life history data along with distribution for this species needs to be collected. The average pH value obtained in this study was in line with the global average and can thus be used as a baseline reading for comparison in future studies. Nudibranchs have the potential to be effective bioindicators of environmental changes as well as reef health. This has been shown through range shifts, along with regional declines in California and Australia. The environmental and experimental factors investigated here did not have an influence on nudibranch abundance, species richness, or composition. Therefore, future experimental procedures to quantify nudibranch populations should not prioritise the influence of the strength of the surge and current, depth, dive time, or number of observers. Future studies can improve their methodologies by increasing the temporal scale of sampling in order to accurately quantify communities and incorporate larger variation within environmental factors. These changes to the sampling procedures will enable long‐term monitoring of nudibranch populations and allow for insight to be gained on the impact of climate change in Sodwana Bay, which may enable nudibranchs to be classed as an effective bioindicator organism.

## AUTHOR CONTRIBUTIONS

**L. Garner:** Conceptualization (lead); data curation (lead); formal analysis (lead); investigation (lead); methodology (lead); software (lead); validation (equal); visualization (lead); writing – original draft (lead); writing – review and editing (equal). **C. J. Oosthuizen:** Conceptualization (supporting); data curation (supporting); formal analysis (supporting); funding acquisition (lead); investigation (supporting); methodology (supporting); project administration (lead); resources (lead); software (supporting); supervision (lead); validation (supporting); visualization (supporting); writing – original draft (supporting); writing – review and editing (supporting).

## FUNDING INFORMATION

None.

## CONFLICT OF INTEREST STATEMENT

There is no conflict of interest.

## Data Availability

All the data has been submitted as part of the supplementary material in the appendices.

## APPENDIX 1

### 1.1

Dive sites sampled for the incidence‐based dataset with their corresponding depths and coordinates.

**Table:**

Dive site	Maximum depth (m)	Coordinates
Pinnacles	13.00	27. 31,152 °S 32. 41184 °E
Four Buoy	16.00	27. 31,147 °S 32. 41182 °E
Chains	17.00	27. 31,682 °S 32. 41228 °E
Caves & Overhangs	17.00	27. 31,588 °S 32. 41110 °E
Anton's	15.00	27. 31,670 °S 32. 41090 °E
Roonies	30.00	27. 52,767 °S 32. 69167 °E
Bikini	21.00	27. 31,678 °S 32. 41360 °E
Stringer	15.00	27. 31,825 °S 32. 40936 °E

## APPENDIX 2

### 2.1

Photographs of all species observed within the SB dataset arranged in alphabetical order. Species names can be found below the corresponding photograph. All images were taken by individuals within the research team.

## APPENDIX 3

### 3.1

A comprehensive list of all the species observed in the sample‐based dataset, including the family to which the species belongs, their abundances at each of the three dive sites, and total abundance. The dive sites samples were Chains (C), Anton's (A), and Bikini (B). Unique species belonging to this dataset are indicated by an asterisk.

**Table:**

Species	Family	Number of individuals per dive site			Total
		C	A	B	
*Ardeadoris angustolutea**	Chromodorididae	0	2	0	2
*Ardeadoris undaurum*	Chromodorididae	1	0	0	1
*Bornella anguilla*	Bornellidae	0	1	0	1
*Ceratosoma* sp. 1*	Chromodorididae	1	4	0	5
*Ceratosoma tenue**	Chromodorididae	0	0	3	3
*Chromodoris africana*	Chromodorididae	0	0	2	2
*Chromodoris annae**	Chromodorididae	0	0	1	1
*Chromodoris celinae*	Chromodorididae	0	2	0	2
*Chromodoris hamiltoni*	Chromodorididae	9	7	7	23
*Dermatobranchus fasciatus**	Arminidae	0	2	0	2
*Dermatobranchus fortunatus**	Arminidae	1	0	0	1
*Dermatobranchus oculus**	Arminidae	2	0	0	2
*Dermatobranchus rodmani**	Arminidae	3	3	0	6
*Dermatobranchus tuberculatus**	Arminidae	1	1	0	2
*Diaphorodoris mitsuii*	Calycidorididae	1	0	0	1
*Doris ananas*	Dorididae	2	1	1	4
*Flabellina flammea*	Flabellinidae	0	1	5	6
*Flabellina* sp. 1	Flabellinidae	0	0	1	1
*Glossodoris acosti**	Chromodorididae	0	0	1	1
*Glossodoris bonwanga*	Chromodorididae	17	10	17	44
*Glossodoris hikuerensis**	Chromodorididae	0	1	0	1
*Glossodoris pallida*	Chromodorididae	1	0	0	1
*Goniobranchus albonares**	Chromodorididae	0	1	0	1
*Goniobranchus albopunctatus*	Chromodorididae	1	1	0	2
*Goniobranchus alius**	Chromodorididae	0	1	0	1
*Goniobranchus* cf *alderi*	Chromodorididae	0	0	1	1
*Goniobranchus conchyliatus**	Chromodorididae	1	0	0	1
*Goniobranchus geminus**	Chromodorididae	0	1	0	1
*Goniobranchus geometricus*	Chromodorididae	0	1	1	2
*Goniobranchus pruna**	Chromodorididae	0	0	1	1
*Goniobranchus tennentanus*	Chromodorididae	0	0	3	3
*Goniobranchus verrieri*	Chromodorididae	0	0	1	1
*Halgerda indotessellata**	Discodorididae	0	0	1	1
*Halgerda toliara*	Discodorididae	1	0	0	1
*Halgerda wasinensis*	Discodorididae	26	10	22	58
*Hexabranchus sanguineus*	Hexabranchidae	2	0	1	3
*Hypselodoris infucata*	Chromodorididae	0	0	1	1
*Hypselodoris maculosa*	Chromodorididae	3	1	1	5
*Hypselodoris maridadilus*	Chromodorididae	1	1	1	3
*Hypselodoris nigrostriata*	Chromodorididae	0	0	3	3
*Hypselodoris pulchella**	Chromodorididae	0	1	0	1
*Hypselodoris regina*	Chromodorididae	1	0	0	1
*Hypselodoris yarae**	Chromodorididae	0	0	1	1
*Mexichromis* sp. 3*	Chromodorididae	0	1	2	3
*Nembrotha aurea*	Polyceridae	3	0	2	5
*Phyllidia coelestis*	Phyllidiidae	1	1	0	2
*Phyllidia marindica*	Phyllidiidae	0	6	0	6
*Phyllidia ocellata*	Phyllidiidae	0	0	3	3
*Phyllidia varicosa*	Phyllidiidae	3	6	2	11
*Phyllidiella meandrina*	Phyllidiidae	3	0	2	5
*Phyllidiella zeylanica*	Phyllidiidae	7	5	2	14
*Phyllidiopsis gemmata*	Phyllidiidae	5	0	1	6
*Phyllidiopsis xishaensis**	Phyllidiidae	0	0	1	1
*Pteraeolidia semperi*	Facelinidae	8	9	0	17
*Samla bicolor**	Samlidae	0	1	0	1
*Tambja* sp. 3*	Polyceridae	0	1	0	1
*Tenellia sibogae*	Trinchesiidae	0	0	4	4
*Tenellia* sp. 6	Trinchesiidae	0	1	0	1
*Thorunna horlogia**	Chromodorididae	1	0	0	1
*Thorunna punicea**	Chromodorididae	0	0	1	1
*Tritoniopsis elegans*	Tritoniidae	1	0	0	1
*Verconia norba*	Chromodorididae	0	2	0	2
*Verconia simplex**	Chromodorididae	1	0	0	1

## APPENDIX 4

### 4.1

A comprehensive list of all the species observed in the incidence‐based dataset. The species names are displayed along with their respective families, the total number of individuals observed as well as at which of dive sites the observations were made. Unique species belonging to this dataset are indicated by an asterisk.

**Table:**

Species	Family	Total number of individuals	Dive sites
*Antonietta* sp. 2*	Facelinidae	1	Bikini
*Ardeadoris undaurum*	Chromodorididae	2	Anton's, Roonies
*Bornella anguilla*	Bornellidae	9	Bikini, Stringer
*Bornella valdae**	Bornellidae	2	Roonies
*Caloria* sp. 5*	Facelinidae	1	Roonies
*Chromodoris africana*	Chromodorididae	10	Anton's, Bikini, Caves & Overhangs, Chains, Four Buoy, Roonies, Stringer
*Chromodoris celinae*	Chromodorididae	20	Anton's, Bikini, Caves & Overhangs, Four Buoy, Pinnacles, Stringer
*Chromodoris hamiltoni*	Chromodorididae	59	Anton's, Bikini, Caves & Overhangs, Chains, Four Buoy, Pinnacles, Roonies, Stringer
*Chromodoris juvenile*	Chromodorididae	1	Bikini
*Chromodoris quadricolour**	Chromodorididae	2	Four Buoy
*Cratena* sp. 2*	Facelinidae	1	Stringer
*Dermatobranchus*	Arminidae	5	Bikini, Stringer
*Diaphorodoris mitsuii**	Onchidorididae	3	Stringer
*Doris ananas*	Dorididae	3	Bikini, Four Buoy, Roonies
*Favorinus tsuruganus**	Facelinidae	1	Bikini
*Flabellina flammea*	Flabellinidae	3	Stringer
*Flabellina lotos**	Flabellinidae	10	Bikini, Caves & Overhangs, Chains, Pinnacles, Stringer
*Flabellina* sp. 1	Flabellinidae	1	Stringer
*Glossodoris bonwanga*	Chromodorididae	29	Anton's, Bikini, Caves & Overhangs, Chains, Four Buoy, Pinnacles, Stringer
*Glossodoris* sp. 1*	Chromodorididae	1	Bikini
*Glossodoris pallida*	Chromodorididae	5	Bikini, Stringer
*Goniobranchus albopunctatus*	Chromodorididae	1	Stringer
*Goniobranchus alderi*	Chromodorididae	1	Bikini
*Goniobranchus annulatus**	Chromodorididae	1	Roonies
*Goniobranchus geometricus*	Chromodorididae	4	Bikini, Stringer
*Goniobranchus tennentanus*	Chromodorididae	1	Bikini
*Goniobranchus verrieri*	Chromodorididae	1	Bikini
*Gymnodoris citrina**	Gymnodorididae	3	Bikini, Stringer
*Halgerda* sp. 6*	Discodorididae	1	Pinnacles
*Halgerda toliara*	Discodorididae	2	Anton's, Caves & Overhangs
*Halgerda wasinensis*	Discodorididae	61	Anton's, Bikini, Caves & Overhangs, Chains, Four Buoy, Pinnacles, Roonies, Stringer
*Hexabranchus sanguineus*	Hexabranchidae	2	Bikini, Stringer
*Hypselodoris fucata**	Chromodorididae	1	Bikini
*Hypselodoris infucata*	Chromodorididae	5	Bikini
*Hypselodoris maculosa*	Chromodorididae	1	Stringer
*Hypselodoris maridadilus*	Chromodorididae	4	Anton's, Pinnacles, Stringer
*Hypselodoris nigrostriata*	Chromodorididae	3	Bikini, Chains, Stringer
*Hypselodoris regina*	Chromodorididae	5	Anton's, Bikini, Caves & Overhangs
*Mexichromis katalexis**	Chromodorididae	4	Bikini
*Nembrotha aurea*	Polyceridae	1	Stringer
*Nembrotha lineolate**	Polyceridae	2	Caves & Overhangs
*Nembrotha* sp. 2*	Polyceridae	1	Bikini
*Phyllidia alyta**	Phyllidiidae	2	Bikini, Pinnacles
*Phyllidia coelestis*	Phyllidiidae	1	Bikini
*Phyllidia exquisite**	Phyllidiidae	2	Caves & Overhangs, Four Buoy
*Phyllidia marindica*	Phyllidiidae	6	Bikini, Stringer
*Phyllidia ocellata*	Phyllidiidae	4	Anton's, Bikini
*Phyllidia varicosa*	Phyllidiidae	11	Anton's, Bikini, Pinnacles, Stringer
*Phyllidiella meandrina*	Phyllidiidae	1	Anton's
*Phyllidiella zeylanica*	Phyllidiidae	13	Anton's, Bikini, Caves & Overhangs, Chains, Four Buoy, Pinnacles, Stringer
*Phyllidiopsis gemmata*	Phyllidiidae	6	Anton's, Bikini, Chains
*Phyllidiopsis phiphiensis**	Phyllidiidae	1	Stringer
*Plocamopherus margaretae**	Polyceridae	1	Stringer
*Pteraeolidia semperi*	Facelinidae	6	Anton's, Chains, Pinnacles, Stringer
*Roboastra gracilis**	Polyceridae	3	Caves & Overhangs, Four Buoy
*Roboastra luteolineata**	Polyceridae	1	Roonies
*Tenellia sibogae*	Trinchesiidae	6	Bikini, Caves & Overhangs
*Tenellia* sp. 6	Trinchesiidae	1	Bikini
*Tritoniopsis elegans*	Tritoniidae	2	Roonies, Stringer
*Verconia norba*	Chromodorididae	1	Stringer

## APPENDIX 5

### 5.1

A table summarising the three biological indices utilised in this study obtained for each sampling event at each dive site: The Shannon–Weaver index, the Simpson dominance index, and the uniformity index. The uniformity index is calculated for the average Shannon–Weaver value for each dive site. The dive site is denoted by the first letter of its name and the sampling event is denoted by a number next to the letter of the dive site. Nudibranch abundance as well as diversity is also depicted for each sampling event at each dive site.

**Table:**

Dive site	Abundance	Species richness	Shannon–Weaver	Simpson	Uniformity
A1	8	5	1.56	0.78	0.62
A2	15	11	2.30	0.89	
A3	21	13	2.47	0.91	
A4	15	8	1.99	0.85	
A5	27	11	2.26	0.88	
B1	15	11	2.34	0.90	0.64
B2	11	8	1.97	0.84	
B3	19	12	2.38	0.90	
B4	27	13	2.27	0.87	
B5	24	13	2.20	0.84	
C1	19	8	1.78	0.77	0.63
C2	15	8	1.86	0.81	
C3	31	19	2.80	0.93	
C4	22	8	1.95	0.84	
C5	21	12	2.27	0.87	
Average			2.16	0.86	0.63

## References

[ece310676-bib-0001] Albright, R. , Takeshita, T. , Koweek, D. A. , Ninokawa, A. , Wolfe, K. , Rivlin, T. , Nebuchina, Y. , Young, J. , & Caldeira, K. (2018). Carbon dioxide addition to coral reef waters suppresses net community calcification. Nature, 555(7697), 516–519. 10.1038/nature25968 29539634

[ece310676-bib-0002] Arey, L. B. (1918). The multiple sensory activities of the so‐called rhinophore of nudibranchs. American Journal of Physiology‐Legacy Content, 46(5), 526–532. 10.1152/ajplegacy.1918.46.5.526

[ece310676-bib-0003] Australian Museum . (2010). Sea Slug Forum . http://www.seaslugforum.net/

[ece310676-bib-0004] Bellwood, D. R. , Hughes, T. P. , Folke, C. , & Nystrom, M. (2004). Confronting the coral reef crisis. Nature, 429, 827–833. 10.1038/nature02691 15215854

[ece310676-bib-0005] Biermann, C. H. , Schinner, G. O. , & Strathmann, R. R. (1992). Influence of solar radiation, microalgal fouling, and current on deposition site and survival of embryos of a dorid nudibranch gastropod. Marine Ecology Progress Series, 86(3), 205–215. 10.1093/mollus/eyi010

[ece310676-bib-0006] Brierley, A. S. , & Kingsford, M. J. (2009). Impacts of climate change on marine organisms and ecosystems. Current Biology, 19(14), R602–R614. 10.1016/j.cub.2009.05.046 19640499

[ece310676-bib-0007] Burn, R. (2015). Nudibranchs and related molluscs. Museum Victoria Publishing.

[ece310676-bib-0008] Churchill, C. K. C. , Valdés, Á. , & Foighil, Ó. D. (2014). Molecular and morphological systematics of neustonic nudibranchs (Mollusca : Gastropoda: Glaucidae: *Glaucus*), with descriptions of three new cryptic species. Invertebrate Systematics, 28(2), 174–195. 10.1071/IS13038

[ece310676-bib-0009] Clark, K. B. (1975). Nudibranch life cycles in the Northwest Atlantic and their relationship to the ecology of fouling communities. Helgoländer Wissenschaftliche Meeresuntersuchungen, 27, 28–69. 10.1007/BF01611686

[ece310676-bib-0010] Clarke, K. R. , & Warwick, R. M. (2001). Changes in marine communities: An approach to statistical analysis and interpretation (2nd ed.). PRIMER‐E.

[ece310676-bib-0011] Cobb, G. (2022). Nudibranch ID Indo Pacific. In.

[ece310676-bib-0012] Cooper, T. F. , Gilmour, J. P. , & Fabricius, K. E. (2009). Bioindicators of changes in water quality on coral reefs: Review and recommendations for monitoring programmes. Coral Reefs, 28(3), 589–606. 10.1007/s00338-009-0512-x

[ece310676-bib-0013] Costello, D. P. (1938). Notes on the breeding habits of the nudibranchs of Monterey bay and vicinity. Journal of Morphology, 63(2), 319–343. 10.1002/jmor.1050630208

[ece310676-bib-0014] Dale, V. H. , & Beyeler, S. C. (2001). Challenges in the development and use of ecological indicators. Ecological Indicators, 1(1), 3–10. 10.1016/S1470-160X(01)00003-6

[ece310676-bib-0015] Dean, L. J. , & Princep, M. R. (2017). The chemistry and chemical ecology of nudibranchs. Natural Products Report, 34, 1359–1390. 10.1039/C7NP00041C 29135002

[ece310676-bib-0016] Debelius, H. (1996). Nudibranchs and sea snails: Indo‐Pacific field guide. IKAN‐Unterwasserarchiv.

[ece310676-bib-0017] Dickinson, J. L. , Shirk, J. , Bonter, D. , Bonney, R. , Crain, R. L. , Martin, J. , … Purcell, K. (2012). The current state of citizen science as a tool for ecological research and public engagement. Frontiers in Ecology and the Environment, 10(6), 291–297. 10.1890/110236

[ece310676-bib-0018] Doney, S. C. , Ruckelshaus, M. , Duffy, J. E. , Barry, J. P. , Chan, F. , English, C. A. , Galindo, H. M. , Grebmeier, J. M. , Hollowed, A. B. , Knowlton, N. , Polovina, J. , Rabalais, N. N. , Sydeman, W. J. , & Talley, L. D. (2012). Climate change impacts on marine ecosystems. The Annual Review of Marine Science, 4, 11–37. 10.1146/annurev-marine-041911-111611 22457967

[ece310676-bib-0019] Dumdei, E. J. , De Silva, E. D. , Andersen, R. J. , Choudhary, M. I. , & Clardy, J. (1989). Chromodorolide A, a rearranged diterpene with a new carbon skeleton from the Indian Ocean nudibranch *Chromodoris cavae* . Journal of the American Chemical Society, 111(7), 2712–2713. 10.1021/ja00189a055

[ece310676-bib-0020] Ekimova, I. , Deart, Y. , Antokhina, T. , Mikhlina, A. , & Schepetov, D. (2022). Stripes matter: Integrative systematics of *Coryphellina rubrolineata* species complex (Gastropoda: Nudibranchia) from Vietnam. Diversity, 14(4), 294. 10.3390/d14040294

[ece310676-bib-0021] Epstein, H. E. , Hallas, J. M. , Johnson, R. F. , Lopez, A. , & Gosliner, T. M. (2019). Reading between the lines: Revealing cryptic species diversity and colour patterns in *Hypselodoris* nudibranchs (Mollusca: Heterobranchia: Chromodorididae). Zoological Journal of the Linnean Society, 186(1), 116–189. 10.1093/zoolinnean/zly048

[ece310676-bib-0022] Fassbender, A. J. , Orr, J. C. , & Dickson, A. G. (2021). Technical note: Interpreting pH changes. Biogeosciences, 18(4), 1407–1415. 10.5194/bg-18-1407-2021

[ece310676-bib-0023] Feely, R. A. , Doney, S. C. , & Cooley, S. (2009). Ocean acidification: Present conditions and future changes in a high‐CO_2_ world. Oceanography, 22, 36–47. 10.5670/oceanog.2009.95

[ece310676-bib-0024] Fritts‐Penniman, A. L. , Gosliner, T. M. , Ngurah Mahardika, G. N. , & Barber, P. H. (2019). Cryptic ecological and geographic diversification in coral‐associated nudibranchs. Molecular Phylogenetics and Evolution, 144, 106698. 10.1016/j.ympev.2019.106698 31812568

[ece310676-bib-0025] Goddard, J. H. R. , Gosliner, T. M. , & Pearse, J. S. (2011). Impacts associated with the recent range shift of the aeolid nudibranch *Phidiana hiltoni* (Mollusca, Opisthobranchia) in California. Marine Biology, 158(5), 1095–1109. 10.1007/s00227-011-1633-7 24391265 PMC3873086

[ece310676-bib-0026] Goddard, J. H. R. , Schaefer, M. C. , Hoover, C. , & Valdés, Á. (2013). Regional extinction of a conspicuous dorid nudibranch (Mollusca: Gastropoda) in California. Marine Biology, 160(6), 1497–1510. 10.1007/s00227-013-2204-x

[ece310676-bib-0027] Gofas, S. (2021). Nudibranchia in mollusca base. https://www.molluscabase.org/aphia.php?p=taxdetails&id=1762.

[ece310676-bib-0028] Gosliner, T. M. , & Fahey, S. J. (2011). Previously undocumented diversity and abundance of cryptic species: A phylogenetic analysis of Indo‐Pacific Arminidae Rafinesque, 1814 (Mollusca: Nudibranchia) with descriptions of 20 new species of *Dermatobranchus* . Zoological Journal of the Linnean Society, 161(2), 245–356. 10.1111/j.1096-3642.2010.00649.x 21527987 PMC3073124

[ece310676-bib-0029] Gosliner, T. M. , Valdés, Á. , & Behrens, D. W. (2015). Nudibranch & sea slug identification – Indo‐Pacific (2nd ed.). New World Publications.

[ece310676-bib-0030] Hoegh‐Guldberg, O. , Mumby, P. , Hooten, A. J. , Steneck, R. S. , Greenfield, P. F. , Gomez, E. , … Hatziolos, M. E. (2007). Coral reefs under rapid climate change and ocean acidification. Science, 318(5857), 1737–1742. 10.1126/science.1152509 18079392

[ece310676-bib-0031] Holt, E. A. , & Miller, S. W. (2011). Bioindicators: Using organisms to measure environmental impacts. Nature Education Knowledge, 2(2), 8.

[ece310676-bib-0032] Ingole, P. D. (2021). Environment and global warming in current scenario. International Journal of Agriculture and Rural Economic Research, 9(6), 16–19. 10.36713/epra0813

[ece310676-bib-0033] Jackson, L. E. , Kurtz, J. C. , & Fisher, W. S. (2000). Evaluation guidelines for ecological indicators. U.S. Environmental Protection Agency.

[ece310676-bib-0034] Johnson, R. F. , & Gosliner, T. M. (2012). Traditional taxonomic groupings mask evolutionary history: A molecular phylogeny and new classification of the Chromodorid nudibranchs. PLoS One, 7(4), e33479. 10.1371/journal.pone.0033479 22506002 PMC3323602

[ece310676-bib-0035] Kara, K. S. L. , Terrence, M. G. , & Nerida, G. W. (2018). Flexible colour patterns obscure identification and mimicry in Indo‐Pacific *Chromodoris* nudibranchs (Gastropoda: Chromodorididae). Journal of Molecular Phylogenetics and Evolution, 124, 27–36. 10.1016/j.ympev.2018.02.008 29476907

[ece310676-bib-0036] King, D. (2014). The Reef Guide: Fishes, corals, nudibranchs & other vertebrates. In East & South Coasts of Southern Africa. Penguin Random House South Africa.

[ece310676-bib-0037] Knowlton, N. , & Jackson, J. B. C. (2008). Shifting baselines, local impacts, and global change on coral reefs. PLoS Biology, 6(2), e54. 10.1371/journal.pbio.0060054 18303956 PMC2253644

[ece310676-bib-0038] Kobori, H. , Dickinson, J. L. , Washitani, I. , Sakurai, R. , Amano, T. , Komatsu, N. , … Miller‐Rushing, A. J. (2016). Citizen science: A new approach to advance ecology, education, and conservation. Ecological Research, 31(1), 1–19. 10.1007/s11284-015-1314-y

[ece310676-bib-0039] Koerner, S. E. , Avolio, M. L. , Chang, C. C. , Gray, J. , Hoover, D. L. , & Smith, M. D. (2015). Invasibility of amesic grassland depends on the time‐scale of fluctuating resources. Journal of Ecology, 103, 1538–1546. 10.1111/1365-2745.12479

[ece310676-bib-0040] Korshunova, T. , Lundin, K. , Malmberg, K. , Picton, B. , & Martynov, A. (2018). First true brackish‐water nudibranch mollusc provides new insights for phylogeny and biogeography and reveals paedomorphosis‐driven evolution. PLoS One, 13(3), e0192177. 10.1371/journal.pone.0192177 29538398 PMC5851531

[ece310676-bib-0041] Krebs, C. J. (1999). Ecological methodology (2nd ed.). Benjamin/Cummings.

[ece310676-bib-0042] Kurnianda, V. , Winahyu, D. A. , Firdaus, R. , Wahyudi, E. , & Musman, M. (2020). Biological and chemical diversity of the Indonesian marine nudibranchs based on MS/MS molecular networking approach. Journal of Aquatic, Coastal and Fishery Sciences, 9(1), 83–94. 10.13170/depik.9.1.15126

[ece310676-bib-0043] Marshall, N. J. , Kleine, D. A. , & Dean, A. J. (2012). CoralWatch: Education, monitoring, and sustainability through citizen science. Frontiers in Ecology and the Environment, 10(6), 332–334. 10.1890/110266

[ece310676-bib-0044] Matsuda, S. B. , & Gosliner, T. M. (2018). Glossing over cryptic species: Descriptions of four new species of *Glossodoris* and three new species of *Doriprismatica* (Nudibranchia: Chromodorididae). Zootaxa, 4444(5), 501–529. 10.11646/zootaxa.4444.5.1 30313904

[ece310676-bib-0045] McGeoch, M. A. (1998). The selection, testing and application of terrestrial insects as bioindicators. Biological Reviews, 73(2), 181–201. 10.1111/j.1469-185X.1997.tb00029.x

[ece310676-bib-0046] McPhail, K. , & Davies‐Coleman, M. T. (1997). New spongiane diterpenes from the east African nudibranch *Chromodoris hamiltoni* . Tetrahedron, 53(13), 4655–4660. 10.1016/S0040-4020(97)00198-1

[ece310676-bib-0047] Miller‐Rushing, A. , Primack, R. , & Bonney, R. (2012). The history of public participation in ecological research. Frontiers in Ecology and the Environment, 10(6), 285–290. 10.1890/110278

[ece310676-bib-0048] Murphy, B. F. , & Hadfield, M. G. (1997). Chemoreception in the nudibranch gastropod *Phestilla sibogae* . Comparative Biochemistry and Physiology Part A: Physiology, 18(3), 727–735. 10.1016/S0300-9629(97)00014-5

[ece310676-bib-0049] Navarro‐Barranco, C. , Ros, M. , de Figueroa, J. M. T. , & Guerra‐García, J. M. (2020). Marine crustaceans as bioindicators: Amphipods as case study. In G. Lovrich & M. Thiel (Eds.), Fisheries and aquaculture (Vol. 9, pp. 435–462). Oxford University Press.

[ece310676-bib-0050] Niemi, G. J. , & McDonald, M. E. (2004). Application of ecological indicators. Annual Review of Ecology, Evolution, and Systematics, 35, 89–111. 10.1146/annurev.ecolsys.35.112202.130132

[ece310676-bib-0051] Niemi, G. J. , Wardrop, D. , Brooks, R. , Anderson, S. , Brady, V. , Paerl, H. , Rakocinski, C. , Brouwer, M. , Levinson, B. , & McDonald, M. (2004). Rationale for a new generation of indicators for coastal waters. Environmental Health Perspectives, 112(9), 979–986. 10.1289/ehp.6903 15198917 PMC1247190

[ece310676-bib-0052] Nimbs, M. J. , Willan, R. C. , & Smith, S. D. A. (2015). Range extensions for heterobranch sea slugs (formerly opisthobranch) belonging to the families Diaphanidae, Plakobranchidae and Facelinidae on the eastern coast of Australia. Marine Biodiversity Records, 8, e76. 10.1017/S1755267215000524

[ece310676-bib-0053] Odum, E. P. (1967). Fundamentals of ecology. Saunders.

[ece310676-bib-0054] Orr, J. C. , Fabry, V. J. , Aumont, O. , Bopp, L. , Doney, S. C. , Feely, R. A. , Gnanadesikan, A. , Gruber, N. , Ishida, A. , Joos, F. , Key, R. M. , Lindsay, K. , Maier‐Reimer, E. , Matear, R. , Monfray, P. , Mouchet, A. , Najjar, R. G. , Plattner, G.‐K. , Rodgers, K. B. , … Yool, A. (2005). Anthropogenic ocean acidification over the twenty‐first century and its impact on calcifying organisms. Nature, 437(7059), 681–686. 10.1038/nature04095 16193043

[ece310676-bib-0055] Paulangan, Y. P. , Supoyo, A. S. , & Kalor, J. D. (2021). Diversity, uniformity and dominance index of nudibranch in Humbolt Bay Water Jayapura City Papua, Indonesia. Journal of Tropical Fisheries Management, 5(1), 59–64. 10.29244/jppt.v5i1.34406

[ece310676-bib-0056] R Core Team . (2021). R: A language and environment for statistical computing. R Foundation for Statistical Computing. https://www.R‐project.org/.

[ece310676-bib-0057] Sabdono, A. , Radjasa, O. K. , Trianto, A. , Sibero, M. T. , Martynov, A. , & Kristiana, R. (2021). An ecological assessment of nudibranch diversity among habitats receiving different degrees of sedimentation in Jepara coastal waters, Indonesia. International Journal of Conservation Science, 12(1), 291–302.

[ece310676-bib-0058] Schleyer, M. H. , & Tomalin, B. J. (2000). Damage on South African coral reefs and an assessment of their sustainable diving capacity using a fisheries approach. Bulletin of Marine Science, 67(3), 1025–1042.

[ece310676-bib-0059] Schultz, S. , Goddard, J. H. R. , Gosliner, T. , Mason, D. , Pence, W. , McDonald, G. , Pearse, V. , & Pearse, J. S. (2011). Climate‐index response profiling indicates larval transport is driving population fluctuations in nudibranch gastropods from the Northeast Pacific Ocean. Limnology and Oceanography, 56(2), 749–763. 10.4319/lo.2011.56.2.0749

[ece310676-bib-0060] Sekizawa, A. , Seki, S. , Tokuzato, M. , Shiga, S. , & Nakashima, Y. (2013). Disposable penis and its replenishment in a simultaneous hermaphrodite. Biology Letters, 9(2), 20121150. 10.1098/rsbl.2012.1150 23407499 PMC3639767

[ece310676-bib-0061] Seroy, S. , & Grünbaum, D. (2018). Individual and population level effects of ocean acidification on a predator‐prey system with inducible defences: Bryozoan‐nudibranch interactions in the Salish Sea. Marine Ecology Progress Series, 607, 1–18. 10.3354/MEPS12793

[ece310676-bib-0062] Shannon, C. E. , & Weaver, W. (1998). The mathematical theory of communication. University of Illinois Press.

[ece310676-bib-0063] Siddig, A. A. , Ellison, A. M. , Ochs, A. , Villar‐Leeman, C. , & Lau, M. K. (2016). How do ecologists select and use indicator species to monitor ecological change? Insights from 14 years of publication in *Ecological Indicators* . Ecological Indicators, 60, 223–230. 10.1016/j.ecolind.2015.06.036

[ece310676-bib-0064] Silvertown, J. (2009). A new dawn for citizen science. Trends in Ecology & Evolution, 24(9), 467–471. 10.1016/j.tree.2009.03.017 19586682

[ece310676-bib-0065] Strö̈mvoll, J. , & Jones, G. (2019). A guide to the sea slugs of the Maputaland. Southern Underwater Research Group.

[ece310676-bib-0066] Swennen, C. (1996). *Gymnodoris Pattani*, a new dorid nudibranch from Pattani Bay, Gulf of Thailand (Gastropoda, Nudibranchia). Bulletin Zoologisch Museum, 15(6), 41–47.

[ece310676-bib-0067] Tibiricá, Y. , Pola, M. , & Cervera, J. L. (2018). Systematics of the genus *Halgerda* Bergh, 1880 (Heterobranchia: Nudibranchia) of Mozambique with descriptions of six new species. Invertebrate Systematics, 32, 1388–1421. 10.1071/IS17095

[ece310676-bib-0068] Tibiriçá, Y. , Pola, M. , Ortigosa, D. , & Cervera, J. (2019). Systematic review of the “*Chromodoris quadricolor* group” of East Africa, with descriptions of two new species of the genus *Chromodoris* Alder & Hancock, 1855 (Heterobranchia, Nudibranchia). Journal of Zoological Systematics and Evolutionary Research, 58(1), 230–261. 10.1111/jzs.12334

[ece310676-bib-0069] Ulfah, M. , Fajri, S. N. , Nasir, M. , Hamsah, K. , & Purnawan, S. (2019). Diversity, evenness and dominance index reef fish in Krueng Raya Water, Aceh Besar. IOP Conference Series: Earth and Environmental Science, 348, 012074. 10.1088/1755-1315/348/1/012074

[ece310676-bib-0070] Wertz, A. , Rossler, W. , Obermayer, M. , & Bickmeyer, U. (2006). Functional neuro‐anatomy of the rhinophore of *Aplysia punctata* . Frontiers in Zoology, 3, 1–11. 10.1186/1742-9994-3-6 16597345 PMC1526719

[ece310676-bib-0071] Wilson, N. , Winters, A. , & Cheney, K. (2016). Tropical range extension for the temperate, endemic South‐Eastern Australian nudibranch *Goniobranchus splendidus* Angas, 1864. Diversity, 8(4), 16. 10.3390/d8030016

